# Triglyceride to high-density lipoprotein cholesterol ratio is an independent predictor of liver fibrosis among pediatrics non-alcoholic fatty liver disease

**DOI:** 10.3389/fendo.2022.1071350

**Published:** 2022-12-16

**Authors:** Yi-Wen Ting, Muhammad Yazid Jalaludin, Azriyanti Anuar Zaini, Rosmawati Mohamed

**Affiliations:** ^1^ Faculty of Medicine, University Malaya, Kuala Lumpur, Malaysia; ^2^ Endocrinology Unit, Department of Pediatrics, Faculty of Medicine, University Malaya, Kuala Lumpur, Malaysia; ^3^ Gastroenterology and Hepatology Unit, Department of Medicine, Faculty of Medicine, University Malaya, Kuala Lumpur, Malaysia

**Keywords:** hepatic fibrosis, type 2 diabetes mellitus, body mass index, overweight and obese children, TG: HDL-C ratio, insulin resistance

## Abstract

**Background:**

Insulin resistance (IR), one of the key components of the metabolic syndrome, is recognized as the pathophysiological hallmark of non-alcoholic fatty liver disease (NAFLD). This study aims to investigate the relationship between surrogate markers of IR and the severity of NAFLD among overweight or obese children.

**Methodology:**

A total of 56 consecutive children aged 6 to 18 years old were recruited from the pediatric obesity and type 2 diabetes mellitus (T2DM) clinic in University Malaya Medical Centre (UMMC) from 2016 to 2019. Data on anthropometric measurements, clinical components of metabolic syndrome and fasting serum insulin were collected. Triglyceride to high-density lipoprotein cholesterol ratio (TG: HDL-C), Homeostatic Model Assessment for Insulin Resistance (HOMA-IR) and Single Point Insulin Sensitivity Estimator (SPISE) were calculated. Transient elastography was performed with hepatic steatosis and liver fibrosis assessed by controlled attenuation parameter (CAP) and liver stiffness measurement (LSM), respectively.

**Results:**

A total of 44 children (78.6%) had liver steatosis and 35.7% had presence of significant liver fibrosis (stage F≥2). Majority (89.3%) are obese and 24 children (42.9%) were diagnosed with metabolic syndrome. Higher number of children with T2DM and significant liver fibrosis were associated with higher tertiles of TG: HDL-C ratio (p<0.05). Top tertile of TG: HDL-C ratio was an independent predictor of liver fibrosis (OR=8.14, 95%CI: 1.24–53.36, p=0.029). ROC analysis showed that the area under the curve (AUC) of HOMA-IR (0.77) and TG: HDL-C ratio (0.71) were greater than that of metabolic syndrome (0.70), T2DM (0.62) and SPISE (0.22). The optimal cut-off values of HOMA-IR and TG: HDL-C ratio for detecting liver fibrosis among children with NAFLD are 5.20 and 1.58, respectively.

**Conclusion:**

Children with NAFLD and higher TG: HDL-C ratio are more likely to have liver fibrosis. TG: HDL-C ratio is a promising tool to risk stratify those with NAFLD who are at risk of developing advanced liver disease.

## Introduction

Non-alcoholic fatty liver disease (NAFLD), a leading cause of chronic liver disease, is conventionally referred as the hepatic manifestation of metabolic syndrome due to its close relationship with cardiovascular risk factors. Insulin resistance (IR), one of the key components of the metabolic syndrome, is recognized as the pathophysiological hallmark of NAFLD (1). As a result, NAFLD is commonly present in up to two thirds of adult with type 2 diabetes mellitus (T2DM) and up to 90% of obese patients (2, 3). Although data among children is scarce, studies have reported nearly 30% of children with NAFLD had T2DM or prediabetes (4) and prevalence of NAFLD in obese children is up to 80% (5). Moreover, early development and increased incidence of T2DM, cardiovascular disease and NAFLD are observed in younger generations due to the worldwide epidemic of childhood obesity (6).

NAFLD consist of a spectrum of disease ranging from steatosis, characterized by fat infiltration in the liver, to a more severe form, non-alcoholic steatohepatitis (NASH), which is the presence of fat infiltration, lobular inflammation, hepatocyte ballooning with or without fibrosis. Increasing fibrosis leads to cirrhosis and liver cancer. Fibrosis stage is the most important prognostic factor in NAFLD (7, 8). Growing literature suggests that cardiovascular disease is the commonest cause of death, followed by malignancy and liver related mortality (9, 10).

Quantifying IR by the gold standard method, hyperinsulinemic euglycemic clamp, in clinical settings is impractical due to its complexity and high cost of test (11, 12). Therefore, surrogate markers of IR, which are more practical and reliable in clinical settings, were utilized. Triglyceride to high-density lipoprotein cholesterol ratio (TG: HDL-C) and Homeostatic Model Assessment for Insulin Resistance (HOMA-IR) have been validated as promising surrogates of IR in various clinical studies (13–16). In addition, recent studies reported that TG: HDL-C ratio is a predictor of various metabolic-related diseases, including NAFLD (17–21). The independent association of TG: HDL-C ratio and incidence of NAFLD was also observed among non-obese individuals without dyslipidemia (22). However, due to the large variability of cut-off values for TG: HDL-C ratio, a refined formula, the Single Point Insulin Sensitivity Estimator (SPISE), was introduced and validated in both adults and adolescents as a new surrogate parameter of IR (23).

The use of IR parameters may be a simple yet powerful tool to identify children with NAFLD who are more likely to develop progressive liver disease. However, existing studies demonstrating the association between TG: HDL-C ratio and the incidence of NAFLD are largely based on the adult population. Hence, the relationship between surrogates of IR and NAFLD among children and adolescents remains to be elucidated. In the present study, we aim to investigate the relationship between surrogate markers of IR and severity of NAFLD among the obese and diabetic children.

## Methods

### Study design and population

This is a cross-sectional study which involved universal sampling method of recruiting 56 consecutive children aged 6 to 18 years old from the pediatric obesity and T2DM clinic in University Malaya Medical Centre (UMMC) from 2016 to 2019. Study subjects of parents who consented were included in the study. These children underwent liver stiffness measurement (LSM) and assessment of liver steatosis by controlled attenuation parameter (CAP) using transient elastography (FibroScan, Echosens, Paris). Exclusion criteria were presence of viral hepatitis, autoimmune hepatitis, history of alcohol consumption, genetic disorders or syndromic children, drug-induced fatty liver, or any other evidence of chronic liver diseases. The study protocol was approved by the UMMC Medical Research Ethics Committee (MREC ID No.: 2019813-7734) and conformed to the provisions of the Declaration of Helsinki. Written informed consent was obtained from parent or legal guardian of children under 18 years of age, two of the 18 years old adolescents provided their own consent.

### Anthropometric measurements and biochemical parameters

Every child was assessed after overnight fasting for at least 8 hours. Anthropometric measurements including body weight, height and waist circumference (WC), and blood pressure (BP) were measured and recorded by trained personnel using standard protocol and equipment. BMI was calculated as weight in kilogram divided by height in meters^2^. Thorough history taking and physical examination were carried out by trained doctors and pediatricians. All participants had venous blood drawn after an overnight fast for fasting blood glucose (FBG), glycated hemoglobin (HbA1c), fasting serum insulin, lipid profile [total cholesterol (TC), serum triglycerides (TG) level, serum high-density lipoprotein cholesterol (HDL-C) level, serum low-density lipoprotein cholesterol (LDL-C) level] and liver enzymes [alanine aminotransferase (ALT), aspartate aminotransferase (AST), gamma-glutamyl transferase (GGT)]. HOMA-IR model was used to quantify IR and calculated by multiplying FBG (mmol/l) and fasting serum insulin (mU/l) divided by 22.5 (16, 24). TG: HDL-C ratio was calculated as serum TG level (mmol/L) divided by HDL-C level (mmol/L). SPISE was calculated with the formula: SPISE = 600 × HDL-C^0.185^/(TG^0.2^ × BMI^1.338^), with HDL-C and TG in mg/dL and BMI in kg/m^2^.

### Definitions

BMI-for-age percentile was calculated using World Health Organization (WHO) growth chart and was classified according to the following; > +1 standard deviation (SD) as overweight, > +2 SD as obesity, and > +3 SD as morbid obesity (25). Central obesity was defined as WC ≥ 90^th^ percentile for age and gender (26). Metabolic syndrome was defined using the International Diabetes Federation (IDF) criteria with presence of central obesity and two or more of the following criteria: serum TG level ≥ 1.7 mmol/L, serum HDL-C < 1.03 mmol/L, systolic BP of ≥ 130 mmHg or diastolic BP of ≥ 85 mmHg and FBG ≥ 5.6 mmol/L or known T2DM (27). Children younger than 10 years old who fulfilled the IDF metabolic syndrome criteria are categorized as at risk of metabolic syndrome (27). HOMA-IR value of ≥ 4.0 indicates IR whereas < 4.0 was considered insulin sensitive (28, 29). Meanwhile HOMA-IR value of >2.6 is used for children <10 years old (pre-pubertal stage). ALT > 25 IU/L was considered abnormal in boys and > 22 IU/L in girls (30).

### Transient elastography (FibroScan) for liver steatosis and fibrosis assessment

Transient elastography (TE) (FibroScan, Echosens, Paris) was performed by a dedicated and specially trained personnel using the 3.5-MHz M probe. An average LSM value and CAP score were obtained after 10 successful measurements. CAP scores were classified into the following: ≤ 248 dB/m (normal), 249–268 dB/m (mild steatosis), 269–280 dB/m (moderate steatosis), and > 280 dB/m (severe steatosis) (31). We considered the cut-offs for LSM values as < 7.0 kPA (F0–1, no significant fibrosis), 7.0–8.6 kPa (F≥2, significant fibrosis), 8.7–10.2 kPa (F≥3, moderate fibrosis), and ≥10.3 kPa (F4, cirrhosis) (32). Presence of steatosis was defined as mild to severe steatosis whereas presence of fibrosis was defined as fibrosis stage F≥2.

### Statistical analysis

Sample size was calculated based on the difference in means of CAP and LSM between obese children and non-obese children. The effect size (Cohen d) for CAP and LSM is 0.92, giving a sample size of 21 (**Supplementary Material**). Data analyses were carried out using SPSS 23.0 (IBM Corp., Chicago, IL, USA). Values were presented as median and interquartile range (IQR) for continuous variables while categorical variables were recorded as number and percentages. All variables were tested for normality using Kolmogorov–Smirnov test. The whole study population was stratified into tertiles of the TG: HDL-C ratio and Kruskal-Wallis test was used to compare medians between the three groups. Univariate and multivariate logistic regression analyses were performed to identify the association of TG: HDL-C ratio, SPISE and other metabolic factors with the presence of liver steatosis and fibrosis. Receiver operating characteristic (ROC) curve analysis was used to calculate area under the ROC curve (AUC) of each IR marker or metabolic syndrome for the presence of liver fibrosis. P value of <0.05 was considered as statistically significant.

## Results

The demographic data and clinical characteristics of all 56 subjects were summarized and published in our previous paper (33). A detailed breakdown of demographic and clinical characteristics of the study population can be found in **Supplementary Table 1**. Overall, the mean age of subjects was 13 (2.8) years old. Sixty percent were males and 66.1% were ethnic Malays. More than three quarter of study population (78.6%) had liver steatosis with 71.4% of them had severe steatosis. Presence of significant liver fibrosis (stage F≥2) was detected in 20 (35.7%) children, of which 10 (17.9%) had fibrosis stage 2, five (8.9%) had fibrosis stage 3 and five (8.9%) had liver cirrhosis or fibrosis stage 4. Majority (89.3%) of the study population are obese, of which, 27 (48.2%) of them are morbidly obese. A total of 24 (42.9%) children were diagnosed with metabolic syndrome.

### Comparison of clinical characteristics according to tertiles of TG: HDL-C ratio

Anthropometric, clinical and metabolic parameters of all subjects are summarized according to tertiles of TG: HDL-C ratio in **Table 1**. Among those in the third tertile of TG: HDL-C ratio, 100% (n = 14) had metabolic syndrome and 50% (n = 7) had T2DM. There was a progressive increase in serum FBG and HbA1c level from the lower to the upper tertile of TG: HDL-C ratio (p=0.049 and p=0.041 respectively). A significantly higher number of children with stage F≥2 liver fibrosis (n = 9, 64.3%) was seen at the third tertile of TG: HDL-C ratio. TG: HDL-C ratio was not significantly associated with HOMA-IR and SPISE.

**Table 1:** Comparison of demographic and clinical characteristics of the study population according to tertiles of the TG: HDL-C ratio.

Characteristics	TG: HDL-C ratio^§^, n = 56			
	Tertile I (n=14)	Tertile II (n=28)	Tertile III (n=14)	P value
Age (years) (mean ± SD)	11.9 ± 3.3	12.8 ± 2.6	13.3 ± 2.6	0.462
Gender, *n (%)*				0.502
Male Female	10 (71.4) 4 (28.6)	16 (57.1) 12 (42.9)	7 (50.0) 7 (50.0)	
Ethnicity, *n (%)*				0.227
Malay Chinese Indian Others	12 (85.7) 0 2 (14.3) 0	16 (57.1) 4 (14.3) 7 (25.0) 1 (3.6)	9 (64.3) 3 (21.4) 1 (7.1) 1 (7.1)	
T2DM, *n (%)*	1 (7.1)	9 (32.1)	7 (50.0)	0.048
Hypertension, *n (%)*	0	3 (10.7)	2 (14.3)	0.379
BMI (kg/m^2^)	30.0 (24.8, 32.5)	29.7 (27.7, 33.8)	29.9 (26.9, 36.1)	0.676
BMI category, *n (%)*				0.843
Overweight Obese Morbid obese	2 (14.3) 6 (42.9) 6 (42.9)	2 (7.1) 12 (42.9) 14 (40.0)	2 (14.3) 5 (35.7) 7 (50.0)	
Waist circumference (cm) Male Female	89.5 (81.3, 97.3) 94.0 (84.3, 101.1) 76.5 (66.1, 91.4)	92.3 (85.4, 105.1) 97.0 (86.5, 108.0) 89.0 (79.0, 92.5)	100.3 (89.0, 112.9) 101.2 (97.1, 112.9) 89.0 (83.4, 116.0)	0.205
Metabolic syndrome, n (%)	0	10 (35.7)	14 (100.0)	–
BP (mmHg)				
Systolic Diastolic	121 (107,126) 67 (62, 77)	119 (114, 125) 65 (59, 72)	116 (112, 130) 66 (63, 73)	0.895 0.730
FBG (mmol/L)	4.7 (4.6, 4.8)	4.9 (4.6, 5.2)	5.3 (4.8, 9.8)	0.049
HbA1c (%)	5.4 (5.1, 5.5)	5.5 (5.2, 5.8)	5.7 (5.4, 8.1)	0.041
Fasting serum insulin (mU/L)	18.6 (11.9, 28.1)	29.4 (18.0, 45.3)	24.8 (22.5, 41.6)	0.181
HOMA-IR	4.2 (3.1, 6.1)	4.9 (3.6, 8.8)	5.8 (4.7, 13.9)	0.235
SPISE	4.5 (4.2, 5.8)	4.7 (3.8, 5.2)	4.3 (3.4, 5.1)	0.299
Total Cholesterol (mmol/L)	4.6 (3.6, 4.9)	4.4 (3.9, 4.9)	5.0 (4.6, 5.8)	0.063
TG (mmol/L)	0.8 (0.7, 0.9)	1.5 (1.1, 1.8)	2.3 (2.1, 2.6)	<0.0001
HDL-C (mmol/L)	1.2 (1.1, 1.4)	1.1 (1.0, 1.2)	0.9 (0.8, 1.0)	<0.0001
LDL-C (mmol/L)	3.0 (2.3, 3.1)	2.6 (2.2, 3.0)	3.0 (2.5, 3.7)	0.273
ALT (U/L)	26.5 (13.8, 45.5)	26.0 (17.3, 44.8)	43.5 (26.5, 82.8)	0.195
AST (U/L)	28.5 (17.5, 35.8)	27.0 (21.0, 33.0)	22.0 (19.0, 45.0)	0.946
GGT (U/L)	24.0 (17.3, 33.5)	22.5 (17.0, 40.0)	35.0 (21.8, 68.5)	0.303
Platelet (10^9^/L)	314.0 (226.0, 438.0)	344.0 (290.0, 376.0)	350.0 (270.0, 387.5)	0.923
CAP score (dB/m)	309.0 (259.0, 339.8)	293.5 (230.0, 321.5)	309.0 (282.8, 348.0)	0.351
Presence of steatosis, *n* (%)	11 (78.6)	20 (71.4)	13 (92.9)	0.287
LSM score (kPa)	4.2 (3.6, 6.7)	6.1 (4.2, 8.2)	7.4 (4.7, 9.2)	0.165
Presence of fibrosis, *n* (%)	2 (14.3)	9 (32.1)	9 (64.3)	0.020

All data are expressed as median (interquartile range) unless specified. All P values were obtained using Kruskal-Wallis test. Significance was assumed when P <0.05.

^§^Tertile I (25th centile), TG: HDL-C ratio <0.88; tertile II (50th centile), TG: HDL-C ratio 0.88-2.10; tertile III (75th centile), TG: HDL-C ratio >2.10.

TG: HDL-C ratio, triglyceride to high-density lipoprotein cholesterol ratio; T2DM, type 2 diabetes mellitus; BMI, body mass index; BP, blood pressure; FBG, fasting blood glucose; HbA1c, hemoglobin A1c; HOMA-IR, Homeostatic Model Assessment for Insulin Resistance; SPISE, Single Point Insulin Sensitivity Estimator; TG, triglyceride; HDL-C, high-density lipoprotein cholesterol; LDL-C, low-density lipoprotein cholesterol; ALT, alanine aminotransferase; AST, aspartate aminotransferase; GGT, gamma-glutamyl transferase; CAP, controlled attenuation parameter; LSM, liver stiffness measurement.

### Relationship between IR markers, liver steatosis and fibrosis

**Figure 1:**
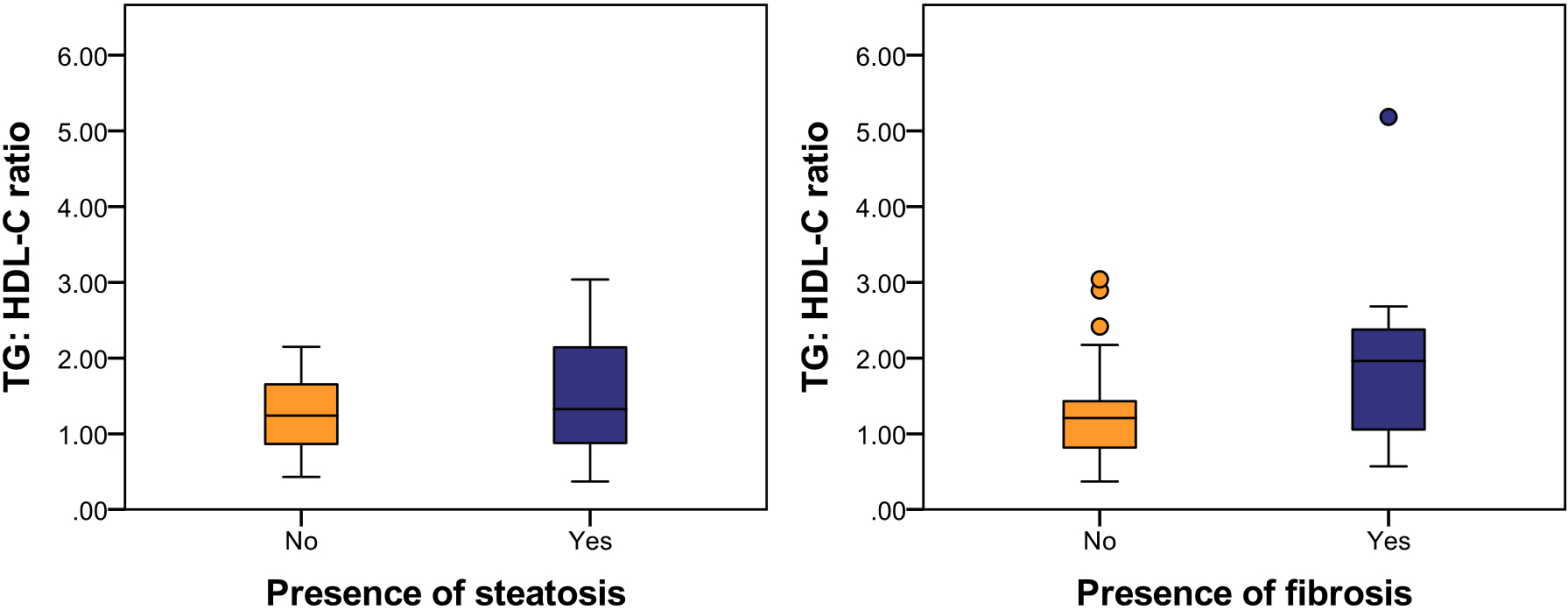
Relationship between TG: HDL-C ratio, liver steatosis and fibrosis. Mann-Whitney U tests were performed and results showed that patients with liver fibrosis have higher TG: HDL-C ratio (p = 0.013) whereas no difference was found among patients with liver steatosis. Significance was assumed when p <0.05.

**Figure 1** illustrates the relationship between TG: HDL-C ratio, liver steatosis and fibrosis. Mann-Whitney U tests were performed and the results showed that patients with presence of liver fibrosis have higher TG: HDL-C ratio (p = 0.013) whereas no significance was found among patients with liver steatosis. In order to investigate the potential independent contribution of TG: HDL-C ratio, HOMA-IR, SPISE and T2DM on liver steatosis and fibrosis, logistic regression analyses were performed (**Tables 2**, **3**). SPISE was associated with presence of liver steatosis and fibrosis after adjusting for age and gender (p < 0.05) but there was no significant association after further adjustments for metabolic factors. Top tertile of TG: HDL-C ratio, HOMA-IR, and T2DM were not associated with liver steatosis (p > 0.05). However, top tertile of TG: HDL-C ratio was a significant predictor of liver fibrosis (OR = 8.14, 95% CI: 1.24 – 53.36, p = 0.029). HOMA-IR and T2DM were associated with liver fibrosis in the univariate analysis but not in the multivariate analysis.

**Table 2:** Odds ratio of insulin resistance parameters and T2DM for liver steatosis in overweight and obese children and adolescents.

Insulin resistance parameters	Model 1	Model 2	Model 3
TG: HDL-C ratio (top tertile)	4.61 (0.54 – 39.49)	4.99 (0.56 – 44.31)	2.66 (0.14 – 52.18)
P value	0.163	0.149	0.52
HOMA-IR ≥ 4	2.16 (0.48 – 9.70)	2.60 (0.48 – 14.17)	1.25 (0.17 – 9.11)
P value	0.315	0.271	0.826
SPISE	0.33 (0.15 – 0.74)	0.21 (0.08 – 0.60)	0.09 (0.01 – 1.85)
P value	0.007	0.003	0.116
T2DM	0.84 (0.21 – 3.28)	0.85 (0.19 – 3.76)	1.39 (0.17 – 11.18)
P value	0.800	0.832	0.756

Logistic regression analysis was used to obtain odds ratio (OR) and P value. Significance was assumed when P <0.05.

Model 1 is univariate analysis; Model 2 is adjusted for age and gender; Model 3 is further adjusted for BMI, metabolic syndrome and presence of fibrosis. (Model 3 for T2DM is further adjusted for BMI and presence of fibrosis only).

TG: HDL-C ratio (top tertile) represents Tertile III.

T2DM, type 2 diabetes mellitus; TG: HDL-C ratio, triglyceride to high-density lipoprotein cholesterol ratio; HOMA-IR, Homeostatic Model Assessment for Insulin Resistance; SPISE, Single Point Insulin Sensitivity Estimator; OR, odds ratio; CI, confidence interval; BMI, body mass index.

**Table 3:** Odds ratio of insulin resistance parameters and T2DM for liver fibrosis in overweight and obese children and adolescents.

Insulin resistance parameters	Model 1	Model 2	Model 3
TG: HDL-C ratio (top tertile)	5.07 (1.39 – 18.46)	5.13 (1.23 – 21.45)	8.14 (1.24 – 53.36)
P value	0.014	0.025	0.029
HOMA-IR ≥ 4	10.11 (1.18 – 86.85)	6.21 (0.65 – 59.09)	4.45 (0.40 – 49.33)
P value	0.035	0.112	0.224
SPISE	0.39 (0.19 – 0.77)	0.46 (0.22 – 0.97)	0.18 (0.02 – 1.86)
P value	0.007	0.042	0.151
T2DM	4.14 (1.24 – 13.81)	2.76 (0.74 – 10.40)	3.35 (0.77 – 14.50)
P value	0.021	0.132	0.106

Logistic regression analysis was used to obtain odds ratio (OR) and P value. Significance was assumed when P <0.05.

Model 1 is univariate analysis; Model 2 is adjusted for age and gender; Model 3 is further adjusted for BMI, metabolic syndrome and presence of steatosis. (Model 3 for T2DM is further adjusted for BMI and presence of steatosis only).

TG: HDL-C ratio (top tertile) represents Tertile III.

T2DM, type 2 diabetes mellitus; TG: HDL-C ratio, triglyceride to high-density lipoprotein cholesterol ratio; HOMA-IR, Homeostatic Model Assessment for Insulin Resistance; SPISE, Single Point Insulin Sensitivity Estimator; OR, odds ratio; CI, confidence interval; BMI, body mass index.

### ROC analysis of IR markers and metabolic syndrome to detect presence of liver fibrosis

**Figure 2:**
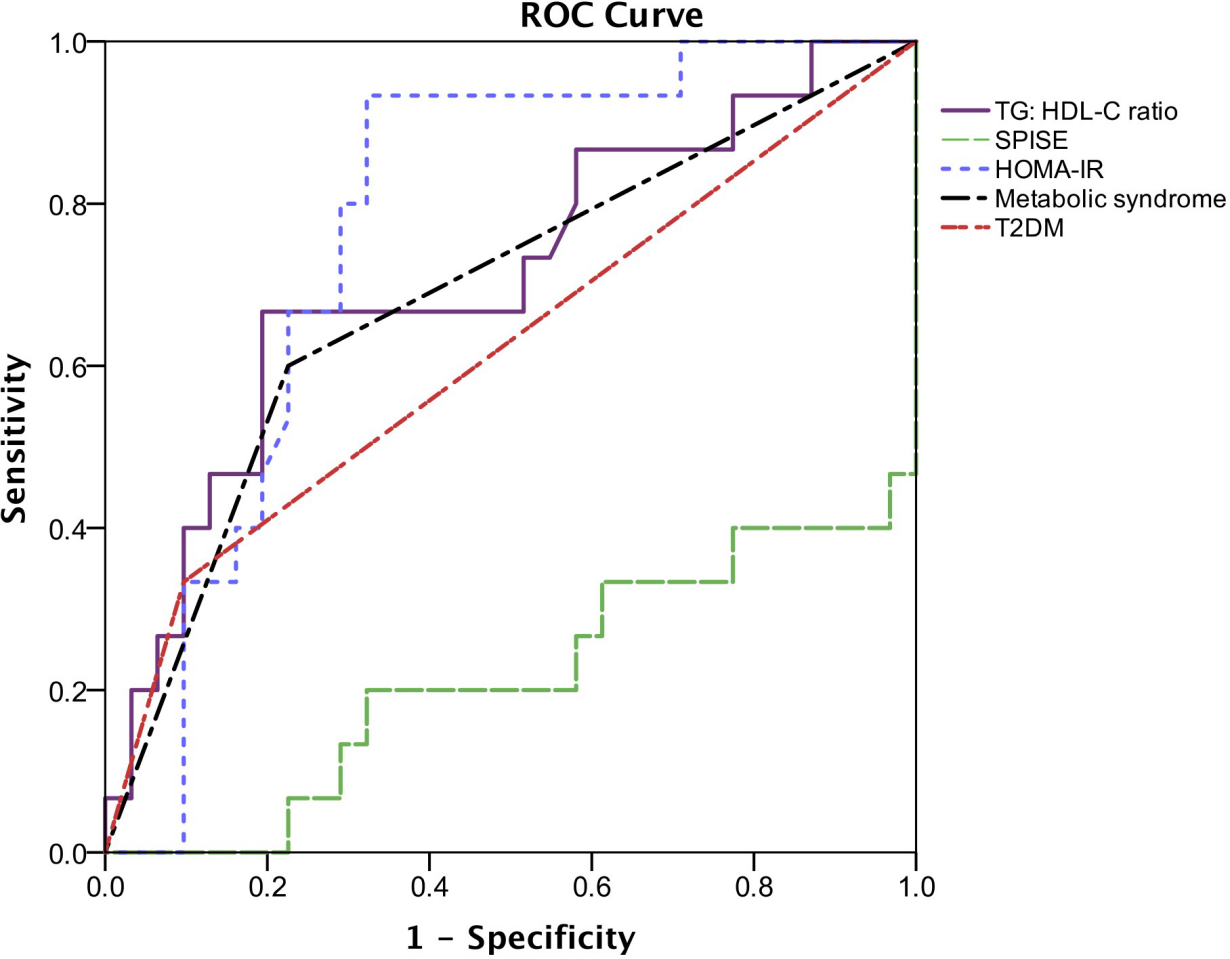
Receiver operating characteristic (ROC) describing the predictive value of insulin resistance parameters, metabolic syndrome and T2DM for the presence of liver fibrosis. The area under the ROC curve (AUC) of TG: HDL-C ratio is 0.71 (95% CI: 0.54-0.88, p = 0.022), SPISE is 0.22 (95% CI: 0.06-0.37, p = 0.002), HOMA-IR is 0.77 (95% CI: 0.63-0.91, p = 0.003), metabolic syndrome is 0.704 (95% CI: 0.54-0.87, p = 0.026) and T2DM is 0.62 (95% CI: 0.44-0.80, p = 0.197).

According to the ROC curves (**Figure 2**), HOMA-IR has the highest AUC (AUC = 0.77, 95% CI: 0.63 – 0.91, p = 0.003), followed by TG: HDL-C ratio and metabolic syndrome (AUC = 0.71, 95% CI: 0.54 – 0.88, p = 0.022 and AUC = 0.70, 95% CI 0.54 – 0.87, p = 0.026, respectively). The AUC of T2DM is 0.62 (95% CI: 0.44-0.80, p = 0.197), whereas SPISE only has an AUC of 0.22 (95% CI: 0.06 – 0.37, p = 0.002). The optimal cut-off point of HOMA-IR for detecting liver fibrosis is 5.20 (sensitivity = 80.0%, specificity = 69.0%) whereas TG: HDL-C ratio with a cut-off value of 1.58 (sensitivity = 67.0%, specificity = 81.0%) is optimal for detecting presence of liver fibrosis.

## Discussion

In recent years, TG: HDL-C ratio is largely utilized as a surrogate marker of IR in both adult and pediatric population. However, racial or ethnic differences in predicting hyperinsulinemia were reported (34). The relationship between TG: HDL-C ratio and IR was first described by McLaughlin and colleagues among 258 overweight or obese adults, which consist of a majority of non-Hispanic whites (15). In Asia, similar results were replicated among Korean population (35) and obese Malay children (36). In contrast, studies have shown that there was no significant association between TG: HDL-C ratio and IR among African Americans and Hispanics (13, 37). In the present study, although we do not find significant association between TG: HDL-C ratio and HOMA-IR among overweight and obese children with NAFLD, this surrogate marker of IR is associated with higher prevalence of T2DM and higher levels of blood glucose parameters. The result is consistent with the finding of TG: HDL-C ratio is positively associated with diabetes risk in the current literature (19, 38). A 15-year prospective study conducted in China found that TG: HDL-C ratio is an independent risk factor of T2DM and that TG: HDL-C ratio is a stronger risk factor than TG (19). Similar conclusion was drawn by another study among Hispanics and African Americans (38). Possible mechanism includes TG: HDL-C ratio as a marker of lipotoxicity in β-cells resulting in reduced insulin secretion (39) and β-cells apoptosis from high triglyceride concentrations (40). Hence, this suggest that the largest effect of the relationship between TG: HDL-C ratio and T2DM may be through β-cell dysfunction apart from the role of IR in T2DM.

Although there is lack of statistical significance, a significant number of children with metabolic syndrome falls into the higher tertile group of TG: HDL-C ratio. Liang et al. reported that TG: HDL-C ratio is a better predictor of metabolic syndrome than the HOMA-IR among the Chinese obese children (41). The anti-lipolytic effect of insulin promotes the accumulation of fat in the body. This effect is worsened in subjects with NAFLD, in which IR occur not only at the level of the muscle but also at the level of the liver and adipose tissue. Moreover, hypertriglyceridemia, as part of atherogenic dyslipidemia, reduces HDL-C level and increases LDL-C level, leading to increased risk of cardiovascular disease (42). A study conducted in Turkey involving 187 obese children and adolescents illustrated that higher HDL-C levels were found in obese children without metabolic syndrome as compared to those with metabolic syndrome, supporting that higher HDL-C levels are preventive factor for metabolic syndrome (43). Therefore, utilizing TG: HDL-C ratio among children may better predict metabolic and cardiovascular risks, especially at the primary care level.

An interesting finding from our study showed that TG: HDL-C ratio is independently associated with significant liver fibrosis (stage F≥2) in children with NAFLD. To the best of our knowledge, existing studies reported significant association between TG: HDL-C ratio and the severity of NAFLD, but none of the studies investigate its association with liver fibrosis. Pacifico et al. summarized that not only is TG: HDL-C ratio a good predictor of cardiovascular disease among children, it is also associated with a higher risk of NAFLD (18). In addition, an adult population-based cohort study in Japan concluded that TG: HDL-C ratio predicts the incidence of NAFLD (21). Subsequent independent studies in Asia have replicated similar findings (17, 22, 44, 45).

There was a lack of observed association between surrogate markers of IR and liver steatosis in this study, but we found that TG: HDL-C ratio is an independent predictor of liver fibrosis. This suggests that TG: HDL-C ratio, as a surrogate marker of IR, may play a significant role in promoting fibrogenesis. Support for the finding that IR is closely related to hepatic fibrosis comes from several studies, in which several mechanisms were proposed for this metabolic disturbance. Firstly, insulin-like growth factor-1 (IGF-1) increases type 1 collagen gene expression and stimulate hepatic stellate cells to proliferate (46). Secondly, IR also impairs the natural killer (NK) cell cytotoxic activity towards activated hepatic stellate cells and promotes the progression of hepatic fibrosis (47). In addition, IR was found to upregulate the expression of profibrotic transforming growth factor-β1 in a dietary rat model (48). These evidences explain the independent association between IR and hepatic fibrosis.

The significant association of TG: HDL-C ratio and liver fibrosis shows that the clinical utility of TG: HDL-C ratio extends beyond identifying patients with IR. Therefore, TG: HDL-C ratio can potentially be utilized as a simple tool for risk stratification to identify overweight or obese children with NAFLD who are at risk of developing advanced liver disease.

Other than TG: HDL-C ratio, T2DM and another two surrogate markers of IR, HOMA-IR and SPISE, were also associated with liver fibrosis. However, these significant associations were diminished after adjusting for confounders. SPISE is a refined TG: HDL-C ratio formula, which includes BMI apart from TG and HDL-C levels, derived from multiple mathematical models and compared to *M*-value derived from euglycemic hyperinsulinemic clamp test (23). Paulmichl and colleagues aimed to enhance the sensitivity and specificity of TG: HDL-C ratio without the use of fasting insulin which is much costly and less available (23). SPISE was shown to have better performance than TG: HDL-C ratio in predicting IR, and performed similarly to HOMA-IR and quantitative insulin sensitivity check index (QUICKI) (23). A study in the North Indian population found that SPISE was significantly lower in patients with metabolic syndrome (49). Similar to TG: HDL-C ratio, SPISE depicts the complex associations of lipoprotein metabolism in the obesity-related cascade. On the other hand, a cross-sectional study involving 361 non-diabetic adults with biopsy-proven NAFLD reported that HOMA-IR was an independent predictor of advanced liver fibrosis (50). The authors have shown that IR contributes to the progression of liver fibrosis independent of BMI (50).

To further investigate the predictive value of surrogate markers of IR to liver fibrosis in obese children with NAFLD, ROC analysis was conducted. In comparison with T2DM and metabolic syndrome, HOMA-IR and TG: HDL-C ratio can better predict liver fibrosis in obese children with NAFLD, whereas SPISE has a significantly lower predictive value. The optimal cut-off points of HOMA-IR and TG: HDL-C ratio to predict presence of significant liver fibrosis were 5.20 and 1.58, respectively. Several independent studies among adult population have reported the use of TG: HDL-C ratio as a predictor of NAFLD (17, 21, 22). The reported optimal cut-off points of TG: HDL-C ratio for incident NAFLD were in the range of 0.65 – 1.4 in men and 0.64 – 0.9 in women. It is expected to have a higher cut-off value for TG: HDL-C ratio to predict the presence of significant liver fibrosis as higher TG: HDL-C ratio is associated with increased risk of progression to advanced liver disease in NAFLD. HOMA-IR is not routinely used in the clinical setting due to the higher cost of fasting serum insulin measurement and less availability in primary healthcare settings. Hence, TG: HDL-C ratio may be a more suitable alternative assessment of severity of NAFLD in obese children. These findings also warrant further investigation in larger population-based study among general pediatric population.

To our knowledge, this study is the first study in South East Asia looking into the relationship between surrogate markers of insulin resistance and hepatic fibrosis in obese pediatric population with NAFLD. This hospital-based study is conducted in a multi-ethnic pediatric population with similar environmental exposure. One of the limitations of our study is that liver biopsy, the gold standard of NAFLD diagnosis, was not performed due to its invasiveness with potential complication risks, and thus, not allowing us to distinguish patients with simple steatosis and non-alcoholic steatohepatitis (NASH). Transient elastography (TE), however, has been widely utilized and validated as a non-invasive assessment tool for liver disease among adult population in recent years (51, 52). Various studies have also validated its use among children, showing comparable results in terms of feasibility and accuracy of TE (53–55). Our study population is relatively small but adequately powered for statistical significance. We were not able to study the effect of TG: HDL-C ratio according to ethnic groups due to the even smaller number of sample size when stratified according to ethnicity.

Recently, a new nomenclature known as the metabolic dysfunction-associated fatty liver disease (MAFLD) has been proposed as an effort in recognizing NAFLD as a standalone disease rather than a diagnosis of exclusion. Diagnosis of MAFLD is based on the histological, imaging or blood biomarker evidence of hepatic steatosis in addition to one of the following three criteria, namely overweight/obesity, presence of T2DM or evidence of metabolic dysregulation (56). While MAFLD has gained remarkable scientific interest in adult population, there is still lack of impact of this newly proposed diagnosis among pediatric population. An argument has raised regarding the less accurate MAFLD diagnostic criteria in a highly selected population such as children with obesity (57). A study published recently also found that the effectiveness of MAFLD diagnostic criteria in identifying the obese children with higher cardiovascular risk is controversial (58). Further researches are needed to better elucidate the utilization of MAFLD diagnostic criteria in the pediatric population.

The early identification of adult patients at increased risk of advanced fibrosis using non-invasive assessment scores such as fibrosis-4 (FIB-4) index and NAFLD fibrosis score allows early intervention. Hence, we recommend the use of TG: HDL-C ratio as a non-invasive assessment to risk stratify children with NAFLD who are at risk of developing advance liver disease.

In conclusion, children with NAFLD and higher TG: HDL-C ratio are more likely to have at least significant liver fibrosis. TG: HDL-C ratio is a promising tool to risk stratify children with NAFLD. Early intervention of the metabolic risk factors can potentially prevent further liver disease progression and avoid adverse clinical outcomes.

## Data availability statement

The datasets presented in this article are not readily available because of relevant data protection laws but are available from the corresponding author on reasonable request within the limitations of informed consent. Requests to access the datasets should be directed to Muhammad Yazid Jalaludin, yazidj@ummc.edu.my.

## Ethics statement

The studies involving human participants were reviewed and approved by University Malaya Medical Centre (UMMC) Medical Research Ethics Committee (MREC ID No.: 2019813-7734). Written informed consent to participate in this study was provided by the participants’ legal guardian/next of kin.

## Author contributions

MJ, AZ, and RM designed the research study. Y-WT, AZ, and MJ established cohort and collected the clinical data for patients. Y-WT carried out data analysis and documented the findings. Y-WT and MJ wrote the manuscript. MJ, AZ, and RM provided critical inputs to the manuscripts. All authors proof read the manuscript. All authors contributed to the article and approved the submitted version.
