# Supplementary material for: Triglyceride to high-density lipoprotein cholesterol ratio is an independent predictor of liver fibrosis among pediatrics non-alcoholic fatty liver disease
Source: Front Endocrinol (Lausanne). 2022 Dec 16;13:1071350. doi: 10.3389/fendo.2022.1071350 (PMC9800858; doi:10.3389/fendo.2022.1071350)
Supplement: Supplementary file 1 [file Table_1.docx]

Supplementary Material

**Sample size calculation from G*Power**

**t tests** - Correlation: Point biserial model

**Analysis**: A priori: Compute required sample size

**Input**: Tail(s) = One

Effect size |ρ| = 0.6

α err prob = 0.05

Power (1-β err prob) = 0.95

**Output**: Noncentrality parameter δ = 3.4369318

Critical t = 1.7291328

Df = 19

Total sample size = 21

Actual power = 0.9521382

**Supplementary Table 1**: Demographic and clinical characteristics of overweight and obese children (n=56).

Note: All data were expressed as median (interquartile range) unless specified.

T2DM, type 2 diabetes mellitus; BMI, body mass index; BP, blood pressure; FBG, fasting blood glucose; HbA1c, haemoglobin A1c; HOMA-IR, Homeostatic Model Assessment for Insulin Resistance; SPISE, Single Point Insulin Sensitivity Estimator; TG, triglyceride; HDL-C, high-density lipoprotein cholesterol; LDL-C, low-density lipoprotein cholesterol; TG: HDL-C ratio, triglyceride to high-density lipoprotein cholesterol ratio; ALT, alanine aminotransferase; AST, aspartate aminotransferase; GGT, gamma-glutamyl transferase; CAP, controlled attenuation parameter; LSM, liver stiffness measurement.

| Variable | All patients (n=56) |
| --- | --- |
| Age (years) (mean ± SD) | 13 ± 2.77 |
| Gender, *n (%)*  Male  Female | 33 (58.9)  23 (41.1) |
| Ethnicity, *n (%)*  Malay  Chinese  Indian  Others | 37 (66.1)  7 (12.5)  10 (17.9)  2 (3.6) |
| T2DM, *n (%)* | 17 (30.4) |
| Hypertension, *n (%)* | 5 (8.9) |
| BMI (kg/m^2^) | 29.8 (27.3, 33.2) |
| BMI category, *n (%)*  Overweight  Obese  Morbid obese | 6 (10.7)  23 (41.1)  27 (48.2) |
| Waist circumference (cm)  Male  Female | 92.3 (84.9, 102.1)  97.0 (87.0, 108.0)  88.0 (79.0, 92.5) |
| Metabolic syndrome, *n (%)* | 24 (42.9) |
| BP (mmHg)  Systolic  Diastolic | 119 (114, 126)  66 (62, 73) |
| FBG (mmol/L) | 4.8 (4.6, 5.3) |
| HbA1c (%) | 5.5 (5.2, 5.8) |
| Fasting serum insulin (mU/L) | 24.8 (17.2, 36.3) |
| HOMA-IR | 4.9 (3.6, 8.1) |
| SPISE | 4.5 (3.9, 5.2) |
| Total Cholesterol (mmol/L) | 4.6 (3.9, 5.0) |
| TG (mmol/L) | 1.5 (0.9, 2.1) |
| HDL-C (mmol/L) | 1.1 (0.9, 1.2) |
| LDL-C (mmol/L) | 2.8 (2.3, 3.1) |
| TG: HDL-C ratio | 1.3 (0.9, 2.1) |
| ALT (U/L) | 28.5 (18.3, 56.8) |
| AST (U/L) | 26.0 (19.5, 36.0) |
| GGT (U/L) | 24.0 (17.3, 42.5) |
| Platelet (10^9^/L) | 344 (270, 381) |
| CAP score (dB/m) | 302 (267, 331) |
| Severity of steatosis, *n* (%)  Normal  Mild  Moderate  Severe | 12 (21.4)  2 (3.6)  2 (3.6)  40 (71.4) |
| LSM score (kPa) | 6.3 (4.0, 8.2) |
| Severity of fibrosis (overall), *n* (%)  No significant fibrosis (F0-1)  Mild (F2)  Moderate (F3)  Severe (F4)  Severity of fibrosis (among patients with steatosis), *n* (%)  No significant fibrosis (F0-1)  Mild (F2)  Moderate (F3)  Severe (F4)  Severity of fibrosis (among patients with metabolic syndrome), *n* (%)  No significant fibrosis (F0-1)  Mild (F2)  Moderate (F3)  Severe (F4) | 36 (64.3)  10 (17.9)  5 (8.9)  5 (8.9)  26 (59.1)  10 (22.7)  4 (9.1)  4 (9.1)  11 (45.8)  8 (33.3)  3 (12.5)  2 (8.3) |
